# Genomic Structural Variations Within Five Continental Populations of *Drosophila melanogaster*

**DOI:** 10.1534/g3.118.200631

**Published:** 2018-08-15

**Authors:** Evan Long, Carrie Evans, John Chaston, Joshua A. Udall

**Affiliations:** * Plant and Wildlife Sciences, Brigham Young University, Provo, UT 84602; † EEOB Department, Iowa State University, Ames, IA, 50011

**Keywords:** Structural Variation (SV), Long-read sequencing, Optical mapping

## Abstract

Chromosomal structural variations (SV) including insertions, deletions, inversions, and translocations occur within the genome and can have a significant effect on organismal phenotype. Some of these effects are caused by structural variations containing genes. Large structural variations represent a significant amount of the genetic diversity within a population. We used a global sampling of *Drosophila melanogaster* (Ithaca, Zimbabwe, Beijing, Tasmania, and Netherlands) to represent diverse populations within the species. We used long-read sequencing and optical mapping technologies to identify SVs in these genomes. Among the five lines examined, we found an average of 2,928 structural variants within these genomes. These structural variations varied greatly in size and location, included many exonic regions, and could impact adaptation and genomic evolution.

Genome structural variations or rearrangements (SV) are thought to play a critical role in plant and animal diversity and speciation. Structural variations are characterized as differences between two aligned genomes that are larger than 50 bp (Alkan *et al.* 2011). Many structural variations can be found among different individuals within the same species (Hardison *et al.* 2003). These variants can include insertions, deletions, duplications, translocations, and inversions (Cao *et al.* 2014). Given their size, they are more likely to disrupt gene function than single-nucleotide variants (SNVs) and significantly contribute to phenotypes and pathology (Lupski 2007). Although many studies refer to SVs as copy number variants (CNVs), the common usage of the term “CNVs” generally applies to a subset of SVs including deletions, insertions, and duplications discovered in short-read resequencing. Because of the short length of reads, the exact nature of the duplications or deletions can remain ambiguous (Zhao *et al.* 2013).

Genome evolution and diversity is often thought to act through the occurrence of SNV. Indeed, SVs have been found to account for 2 to fourfold greater locus-specific mutation frequency than single nucleotide polymorphisms (Redon *et al.* 2006; Lupski 2007). This implies that on average more base pairs are changed through structural variation than by point mutations (Alkan *et al.* 2011). Although researchers are finding an increased appreciation for SVs (Stankiewicz and Lupski 2010; Massouras *et al.* 2012), significant limitations remain for SV detection using short sequencing reads (Zhao *et al.* 2013). Because most SV detection methods involve mapping relatively short reads to a reference genome assembly, the algorithms for SV detection are generally limited to SVs smaller than the read length (Cao *et al.* 2014). This is especially true with respect to insertions, as the algorithms favor calling deletions (Cao *et al.* 2014). Such assumptions may affect signals in phylogenomic analyses if SVs on a varying scales contribute to the genetic diversity between species.

Unlike with short-read sequencing, long-read technologies that span SVs of interest are able to provide genomic resolution for large SVs, especially in repetitive regions. Chromosomal rearrangements are more common in repetitive areas, which pose difficulties to short-read SV detection (Alkan *et al.* 2011). To overcome this limitation and to investigate SVs on a novel scale, we used PacBio long read sequencing combined with BioNano optical mapping to assess SVs across five different *Drosophila melanogaster* lines.

*D. melanogaster* originated on the African continent ∼5.4 million years ago and is now ubiquitous across the globe (Tamura *et al.* 2003; Grenier *et al.* 2015). Its large population size and short generation time enable interesting intraspecies comparisons of flies derived from diverse geographic locations (Tamura *et al.* 2003; Grenier *et al.* 2015). This makes it an ideal model for research in systems biology and population diversity. Using representatives of *D. melanogaster* collected on five different continents to represent diverse lines from around the globe (Table 1), we present 5 high quality genome assemblies using long read sequencing paired with optical maps. We also assessed the diversity of chromosomal structural variations and their potential impacts. We chose this panel within a single Drosophila species because we were primarily interested in intra-species diversity. Several reports have previously compared genomes between different species, but few reports to date have compared more than two *de novo* genome assemblies and assessed intraspecific variation. In addition, low-pass Illumina sequencing had previously been used to quantify inter-specific genetic variation in this particular intra-specific panel of Drosophila (Alkan *et al.* 2011; Grenier *et al.* 2015). Most of the assembled eukaryotic genomes consist of only one *de novo* assembly (Kitts *et al.* 2016). Having multiple assemblies of the same species illuminates the prevalence of genomic structural variation within a species (Alkan *et al.* 2011)(Chakraborty *et al.* 2018). Our study provides insights into the evolution of chromosomal architecture within *D. melanogaster* and sets a benchmark of genome evolution for other comparisons intra-specific genetic variation.

**Table 1:** Assembly statistics for sequence and optical map assemblies of the five global lines of *D. melanogaster*. Optical map alignment is evaluated against the assembled portion of the *D. melanogaster* ISO1 release 5 reference genome

	B59	I23	N25	T29A	ZH26
Location	Beijing (China)	Ithaca (New York)	Netherlands	Tasmania	Zimbabwe
Sequence Assembly Length (Mb)	121.3	115.4	120.0	120.2	117.1
# Of Contigs	38	126	35	24	75
Contig N50 (Mb)	7.56	1.34	5.67	11.4	2.45
BUSCO %	95.7	85.3	96.9	96.3	92.0
Optical Map Length (Mb)	138.2	130.1	166.3	148.5	144.1
Optical Map N50 (Mb)	1.01	0.897	1.255	1.144	1.297
Alignment %	93.9	91.4	90.8	94	92.8
Hybrid Scaffold Length (Mb)	122.0	117.4	120.6	120.3	118.3
# Of Scaffolds	17	38	16	10	31
Scaffold N50 (Mb)	10.24	6.14	8.67	21.47	9.23

## Methods

### Optical Mapping DNA extraction

Before the extraction the flies were starved for 2 hr to reduce the number of reads that would be obtained from gut-associated bacteria. High molecular weight DNA was extracted from adult *D. melanogaster* by first grinding ∼100-200 whole flies to a rough powder with a mortar and pestle in liquid nitrogen. The powder was suspended in homogenization buffer (10 mM Tris HCl pH 7.5, 60 mM NaCl, 10 mM EDTA, 5% sucrose) and disrupted with a 40 mL Dounce homogenizer before filtering through a 100 micrometer (VWR cat. # 21008-949) and 40 micrometer (VWR cat. # 21008-950) nylon mesh sequentially. The resulting pellet was resuspended in 200 uL of resuspension buffer (10 mM Tris HCl pH 7.5, 60 mM NaCl, 10 mM EDTA) and combined with 2% low melting agarose. The mixture was aliquoted into 80 uL plugs and placed in a 4° fridge until solid. The agarose plugs were incubated with 200 µL proteinase K (QIAGEN, cat. # 158920) and 2.5 mL lysate solution (BioNano Prep Lysis Buffer, 20255) overnight and treated with RNase A (QIAGEN, cat. # 158924, 80 µL/mL) as described in BioNano protocol documentation (BioNano Prep Blood DNA Isolation Protocol, Document Number: 30033). DNA was extracted from the agarose plugs by melting and treating the plugs with agarase (Bio-Rad, cat. # 1703594).

### SMRT DNA Extraction

We obtained high molecular weight DNA for single molecule real-time (SMRT) sequencing using a Qiagen genome-tip kit (Cat No./ID: 10243), because the previously explained method could not provide sufficient quantity. We used a modified a extraction protocol outlined in a previous study (Chakraborty *et al.* 2016). First, ∼200 adult flies were ground in liquid nitrogen and transferred into 9.5 mL of buffer G2 with 38 µL of RNAse A (100 mg/ml) and 500 µL of proteinase K (QIAGEN, cat. # 158920). The solution was then incubated overnight at 50°. It was then centrifuged at 5000 × g for 10 min at 4°. The solution was then purified, washed, and eluted using the Qiagen genome-tip kit instructions. Sequencing libraries were created by shearing DNA to 35 kb on a Megaruptor (Diagenode) and selecting for 18-50 kb using a Blue-Pippin (Blue Pippin system, Sage Science, Beverly, MA, USA). DNA was then sequenced using a Sequel machine (Pacific Biosciences, Inc.). We did not include any technical replicates. Data are publicly available and can be found through NCBI under SRA accession SRP142531.

### Assembly and Scaffolding

**Figure 1:**
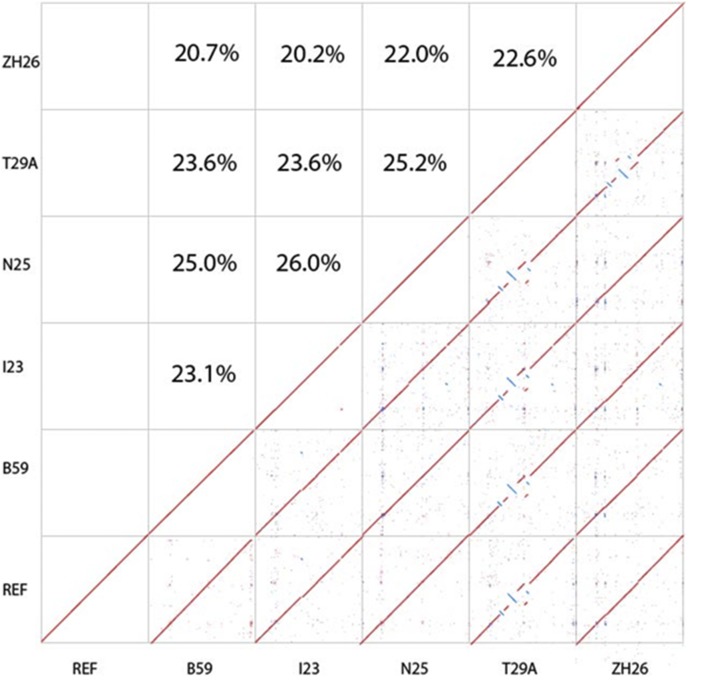
Whole genome alignments between the five global lines and the reference genome of *D. melanogaster*. The X-axis represents each genome used as the reference for alignment by the other lines. Alignment is shown in order chromosome arms 2L, 2R, 3L, 3R, 4, and X. Percentages represent amount of SV coincidence between each of the lines.

PacBio reads were assembled using CANU assembler V1.4. Assemblies were then scaffolded using optical maps with the Solve hybrid scaffold pipeline created by BioNano Genomics. For whole genome collinearity analyses (Figure 1) genomes were scaffolded into whole chromosome arms using the reference genome and the Solve hybrid scaffold pipeline with a minimum alignment p-value of 1E-10. Only contigs that were retained by Bionano hybrid-scaffolding with optical map data and the reference genome continued on for variant analysis. This was done to reduce redundant variant calling due to residual heterozygosity (Grenier *et al.* 2015). The contigs retained by their corresponding scaffolded assemblies were aligned using “mummer” version 3.23 and uploaded to “Assemblytics” (Nattestad and Schatz 2016) for alignment to the reference genome and detection of structural variants (Kurtz *et al.* 2004, File S1, S7). A minimum match of 500 base pairs is required for a single match and 100 for a cluster of matches.

### Annotation and Analysis of Structural Variation

To evaluate the structural evolution in the populations of *D. melanogaster* we analyzed the coincidence of structural variations with other genomic features. This was done by primarily using “IntersectBed” of bedtools (File S7) (Quinlan and Hall 2010). Whole genome alignments were created using minimap2 and minidot (File S7) (Li 2016). By comparing the start and end positions and the identity of each SV in each line we created a relationship matrix relating all five lines. The method for this evaluation permitted 3 base pairs difference in positions to account for any small, secondary differences in genome position and the code can be found in the supplementary files (File S7). We evaluated the evolutionary distance between the lines using the “pvclust” package in R (Figure 4, File S7) (Suzuki and Shimodaira 2006). Each genome was also annotated using GENSAS web based annotation software (File S2, Humann *et al.*).

### Optical Mapping

To visualize the DNA molecules each sample underwent a labeling process that marks a specific hexameric sequence recognized by the restriction enzyme *BssS*I, along each DNA strand. Each molecule was nicked by *BssSI*, labeled with fluorescently labeled nucleotides, repaired to prevent breakage, and counterstained. The process is described in detail in BioNano protocol documentation (BioNano Prep Labeling - NLRS Protocol, Document Number: 30024). The samples were then loaded into flow cells where each individual DNA molecule was moved through nanochannels using electrophoresis and their fluorescence was imaged. We generated an average of four datasets from a single sample of each line, each dataset containing 30 stitched images.

The fragment size data from each DNA molecule were estimated from images using BioNano software. The software estimated the ordered numerical distances between each pair of fluorescent labels on each DNA molecule in the images. Since each molecule represented a ‘unique’ order fragment sizes, individual molecule patterns could be assembled based on overlapping fragment patterns. A BioNano assembly for each line was created using over 100X coverage of molecules with minimum length of 150 kb (File S3). The assemblies were aligned with the published *Drosophila melanogaster* version 5 reference genome for identification of structural variations using BioNano SVdetect (Mak *et al.* 2016).

### Data Availability

Supplemental files are available at FigShare. File S1 contains structural variants reported by Assemblyitics. File S2 contains GENSAS annotations of each genome. File S3 contains optical maps and structural variants detected by optical map alignment to the reference genome. File S4 contains genes affected by structural variation in each line (Heberle *et al.* 2015). File S5 is a table of structural variant found in all 5 lines. File S6 contains sequence assemblies for each line. File S7 contains supplemental methods and parameters. Sequence data are available at NCBI with the SRA accession number: SRP142531. Supplemental material available at Figshare: https://doi.org/10.25387/g3.6203300.

## Results

### Genome Assemblies

We created high-quality sequence, optical map, and hybrid assemblies for each of the five global lines of *D. melanogaster* (Table 1). The estimated genome size of *D. melanogaster* is around 180 Mb, with one-third composed of highly repetitive, heterochromatic sequence and two-thirds of the genome representing the euchromatic regions (Adams *et al.* 2000). In this study, the hybrid assemblies (sequence contigs scaffolded with optical map data) were the most contiguous and represent our final efforts with each line (Table 1). The assembly of line T29A represents the most contiguous genome assembly. The lower contiguity of the I23 and ZH26 assemblies were due to varied sequencing throughput and residual heterozygosity, respectively. With the large majority of SVs detected among the lines being <50kb, a contig N50 of >1Mb is sufficient for variant detection. Each genome has a high BUSCO score validating completeness of the genomes by detecting the presence of widely conserved orthologous genes. Whole genome alignments between each line display the collinearity and completion of each assembly (Figure 1). The large amount of collinearity across the chromosome arms between the reference genome and each assembly confirms between the reference genome and each assembly the likelihood of correctly assembled genomes.

The large contiguity of our assemblies was created by initially assembling sequence contigs from PacBio data. The sequence assemblies were then scaffolded into near pseudomolecule length scaffolds using BioNano optical maps. The number of scaffolds range from 10-38 in each line, so that each of the 4 chromosomes of *D. melanogaster* genome are contained with a small number of scaffolds. The high-quality of these assemblies, shown by their high contiguity and alignment, allowed us to confidently perform further analyses of genomic variation between lines.

The raw assembly of ZH26 contained several putative regions of residual heterozygosity that previous studies have correlated with the presence of chromosomal inversions in other lines. These correlated inversions persisted as heterozygous blocks within inbred lines (Grenier *et al.* 2015). We omitted these heterozygous regions to reduce the chance of calling redundant variants, which may result in an underestimation of structural variation.

### Chromosome Structural Variation

**Figure 2:**
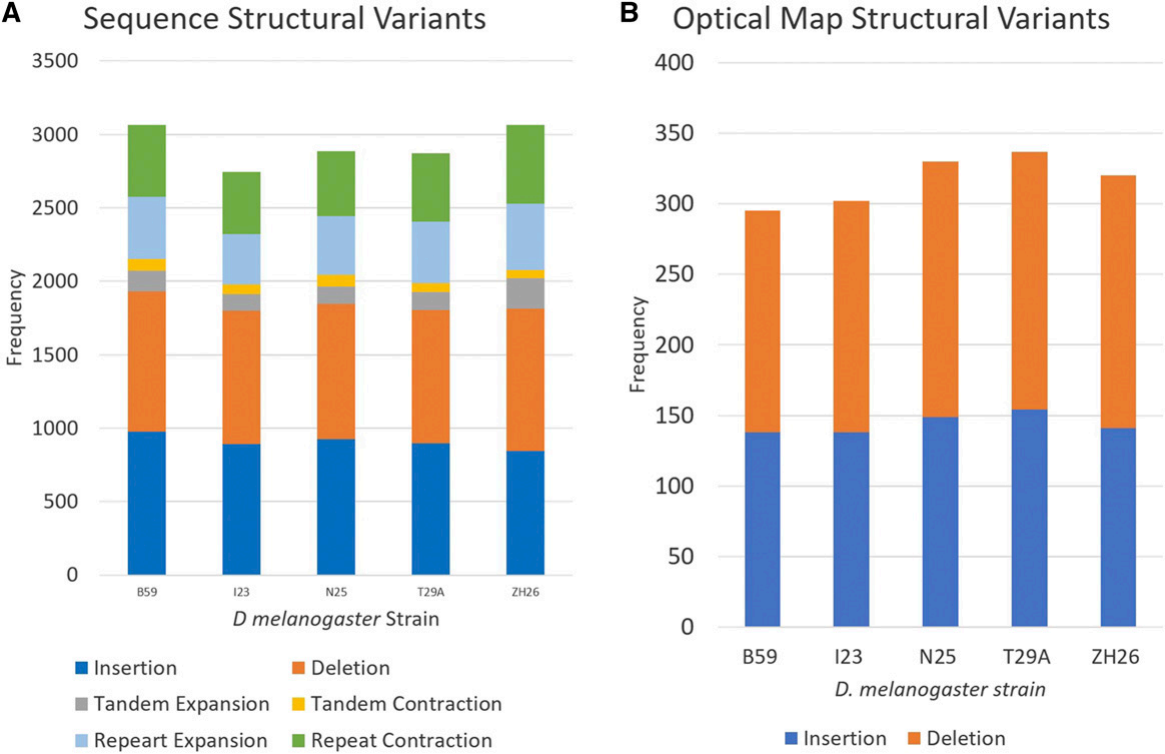
Structural Variant statistics of the five global lines of *D. melanogaster*. A) Classification and frequency of sequence based structural variants called by “Assemblyitics”. B) Classification and frequency of optical map based structural variations called by BioNano SVdetect”.

**Figure 3:**
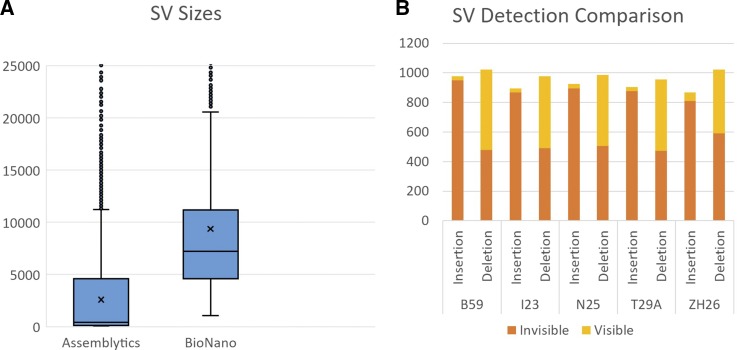
A) Size distribution of structural variants called by both long-read sequencing and Optical Map methods. The Y-axis is defined to show majority of variance and does not display some larger SVs detected. B) Sequence SVs detected by long-read sequencing, but not by short read resequencing are classified as “invisible”.

Both sequence assemblies and optical maps were aligned to the *D. melanogaster* release 5 reference genome for the detection of structural variations (Smith *et al.* 2007). We used this older version of the reference to make chromosome positions consistent with previous work in these lines of *D. melanogaster*. In all our analyses structural variations are defined as discrepancies >50 bp between the assembly and the reference. Structural rearrangements were detected using both sequence alignment and optical map alignment methods (Figure 2A). The “Assemblytics” software classified SVs into insertions, deletions, tandem expansions, tandem contractions, repeat contraction, and repeat expansion (Nattestad and Schatz 2016). We found an average of ∼2,928 SVs in each line (Figure 2A). Of each lines’ SVs, 20–30% were shared with each other line (Figure 1). We also found that 201 of these SVs were shared among all of the 5 lines, indicating a relative uniqueness to the reference genome. It reported a higher frequency of insertions and deletions not associated with tandem or repetitive elements. We also compared the long-read sequence SVs to the previously performed short-read SV detection [10], and found that many of the long-read SVs were undetected using short-read sequencing, especially in regards to insertions (Figure 3B).

**Figure 4:**
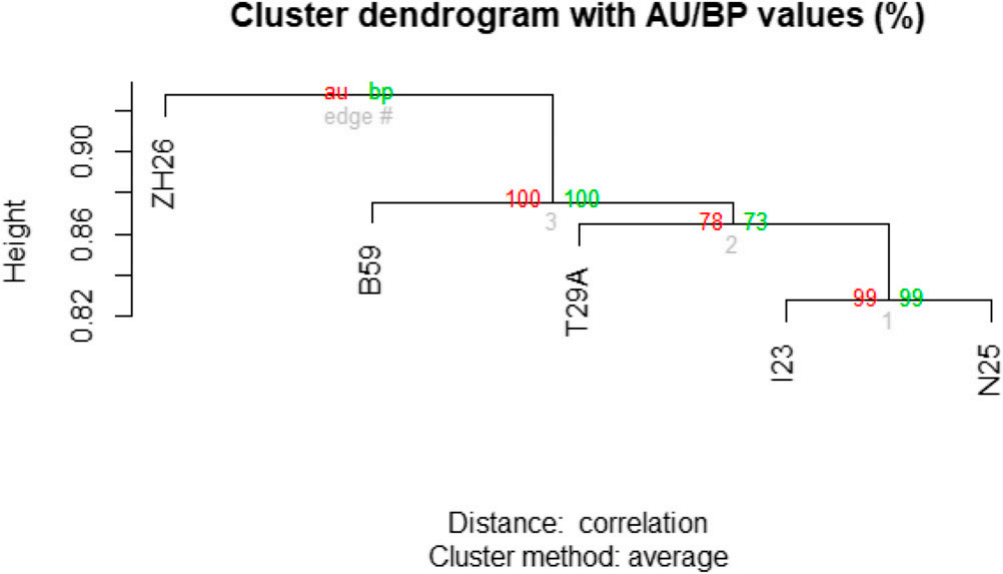
Evolutionary relationships based on coincidence of sequence structural variants between lines. The tree was created using pvclust package in R. Two types of p-values are shown for each branch node: Approximately Unbiased (AU) and Bootstrap Probability (BP).

The optical map alignment detected insertions and deletions independently from the sequence alignment, and found an average of 300 SVs in each line (Figure 2B). The ideal resolution of optical mapping allows for the detection of large SVs (>1000 bp) (Figure 3A) (Cao *et al.* 2014). Both SV detection methods display a balanced frequency between insertions and deletions, a difficult feat by short read sequencing alone which favors calling deletions (Zhao *et al.* 2013). We also created SV density plots across each chromosome arm to visually inspect patterns in SV location distribution. (Figure S2). By examining the coincidence of SVs between each line, we were able to build a relationship tree (Figure 4). This tree places ZH26 as the most differentiated from the other lines.

**Figure 5:**
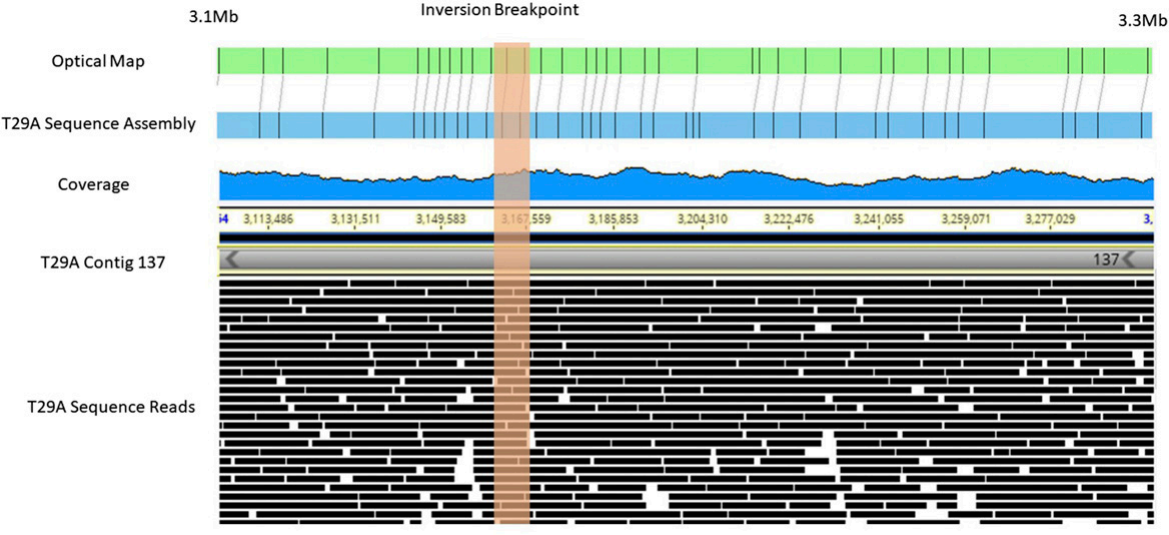
Visualization of region of putative inversion in arm 3R of line T29A relative to the reference genome. Orange vertical bar indicated the breakpoint of the inversion. The optical map alignment is on top (green). PacBio reads are shown along the bottom with coverage shown on a scale from 0-69. Image is merged between BioNano Irysview and Geneious softaware (Kearse *et al.* 2012).

Whole genome alignments revealed large variations (>10kb) including inversions and translocations (Figure 1). Most visible are the large inversions and translocations located on chromosomes 2 and 3 of T29A. To validate the presence of these chromosome rearrangements we aligned reads, contigs, and optical maps to the breakpoints for visual verification (Figure 5), which showed no drop in coverage or contiguity around the proposed inversion breakpoint. The raw assembly of ZH26 showed many residual heterozygous blocks that may be indicative of chromosomal inversions (Grenier *et al.* 2015).

### Genome Evolution

**Figure 6:**
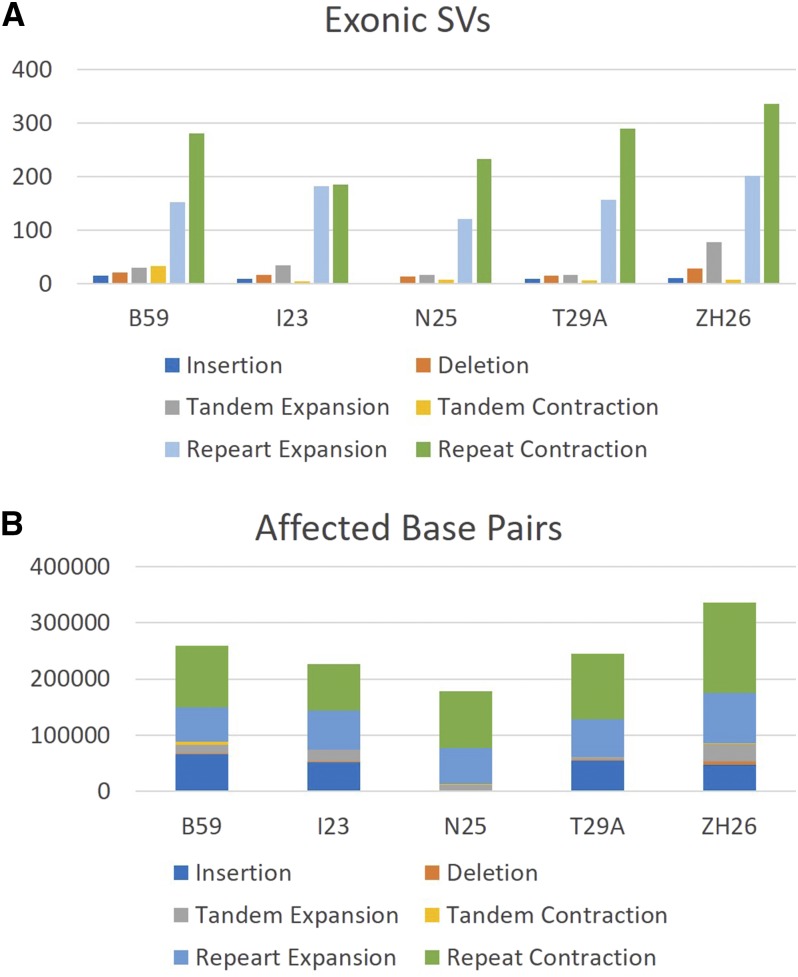
Exons containing Structural Variants in the five global lines of *D. melanogaster*. A) Classification and frequency of structural variants within exonic regions of the genome. B) Total amount of base pairs within exonic regions of the genome affected by structural variants.

We next evaluated the extent to which these structural variations affected the exonic regions of genome. To ensure the accurate calling of these SVs, we only used SVs that were independently validated by the optical map SV detection (Figure S1). There were on average 503 exons overlapping with SVs in each line (Figure 6A, File S4). We calculated the number of base pairs affected by each class of SV (Figure 6B). This total length of exonic sequence affected by SVs was greater than SNVs found in these lines (Grenier *et al.* 2015).

Although there were more insertions and deletions overall, the exons more often contained repeat contractions or expansions SV than the other types of SVs.

## Discussion

In this study we assessed the genetic diversity of structural variations within a single species (*D. melanogaster*) using long-read PacBio sequencing paired with optical mapping. This allowed us to find large structural variations that were previously undetected by short-read sequencing and accurately assess the amount of intra-specific genomic evolution in a globally distributed species. Other studies have been done which describe the relative visibility of these SVs to short read sequencing, showing a strong advantage for long-read over short-read sequencing (Chakraborty *et al.* 2018). We claim a high confidence in the observed structural variation coincidence with exonic regions of the genome, because of the independent identification derived from the high molecular weight DNA sequencing and optical mapping methods. The number of exonic regions of the genome affected by SVs leads us to predict that these SVs are contributing to real evolutionary variation between these lines.

We report a high frequency and variability of chromosomal structural rearrangements within the *D. melanogaster* species across five continental populations. Among the high variability in structural rearrangements we found a many SVs coinciding with gene coding regions of the genome (File S4). Of the 28 genes coinciding with SVs in all 5 lines, MRP, Nop60B, Prosalpha6, and su(w[a]) have been previously implicated in dosage dependent pathways (Hallacli *et al.* 2012; Cugusi *et al.* 2015). Additionally, genes Ugt86Dh, Cyp28d1, and Cyp6a17, previously found to be connected to dosage pathways of nicotine resistance coincide with SVs in more than one of the five lines (Chakraborty *et al.* 2018). Perhaps our SVs are affecting these same pathways contributing to physiological differences and evolutionary divergence between lines.

Specific investigations have assessed the impact of SVs on the divergence and evolution of species (Feulner and De-Kayne 2017). Previous work has shown the retention of SVs to be due to either genetic drift or positive selection. Although we expect that some of the SVs presented here could be the product of positive selection, it remains to be formally tested (Cardoso-Moreira *et al.* 2016). Ideally, these populations would be introduced into each of the respective environments and each line assessed for fitness within specific environmental ranges. Chromosomal rearrangements that impact genes provide testable hypotheses with respect to mechanisms of positive selection, and direct functional tests of gene expression level and consequence phenotypic impact can be relatively straightforward. The alteration of gene number by SVs has been associated with speciation in Drosophila (Ting *et al.* 2004).

Some SVs such as inversions may not directly change the exon or the regulatory sequence of a gene. Although inversions are less likely to have a genic effect, they can influence the recombination between species and create reproductive isolation (Noor *et al.* 2001). The global setting for these lines gives important adaptive context to these SVs. It is more likely for species undergoing migration to contain few variants of large effect (Yeaman and Whitlock 2011). While the putative inversions in T29A and ZH26 could be the initial steps of speciation between two lines of *Drosophila*, previous studies have observed no obvious reduction in fitness of F_1_ individuals resulting from crosses between them and the other lines (Greenberg *et al.* 2010). With the development of newer sequencing and optical mapping technologies, it may be possible to resolve such large rearrangements. For example, the recently released BioNano labeling technology does not use enzymatic knicking that previously limited optical map length.

The consistency between the evolutionary relationships found in our SV coincidence data (Figure 4) and previous work suggests a regular frequency of SVs in *Drosophila* (Grenier *et al.* 2015). Using expected mutation frequencies (Tamura *et al.* 2003), previously produced SNV data (Grenier *et al.* 2015), and our SV data, we postulate that SVs in *D. melanogaster* occur at a rate of ∼50/MY/Mb. The presence of 201 shared SVs across all 5 lines may indicate either SVs that are unique to the reference genome or putative assembly errors in the reference. Though the improvement of the *D. melanogaster* reference genome is not the purpose of this study, researchers may benefit from these genome assemblies knowing that SVs may be present (File S5).

Structural variations constitute a substantial amount of diversity within a species and have an impact on species evolution and genomic divergence that has heretofore been unobserved and under-appreciated. Obtaining multiple *de novo* assemblies of a species allows for the detection of large genomic variations invisible to short-read sequencing. The large size and diversity of these SVs within a *D. melanogaster* suggests that SVs contribute to the genetic diversity of a species and its adaptation to environmental cues. Further studies into the patterns of structural variation could serve to discover the extent of this evolutionary impact.
